# Potential Application of Pyroptosis in Kidney Renal Clear Cell Carcinoma Immunotherapy and Targeted Therapy

**DOI:** 10.3389/fphar.2022.918647

**Published:** 2022-06-15

**Authors:** Xiaochen Qi, Xiangyu Che, Quanlin Li, Qifei Wang, Guangzhen Wu

**Affiliations:** Department of Urology, First Affiliated Hospital, Dalian Medical University, Dalian, China

**Keywords:** KIRC, RCD, pyroptosis, bioinformatics analysis, prognostic model

## Abstract

Renal cell carcinoma (RCC) is a type of cancer with an increasing rate of morbidity and mortality and is a serious threat to human health. The treatment of RCC, especially kidney renal clear cell carcinoma (KIRC), has always been the focus of clinical treatment. Using The Cancer Genome Atlas (TCGA) database as a starting point, we explored the feasibility of applying the pyroptosis mechanism to KIRC treatment by searching for cancer markers associated with pyroptosis and cancer treatment signatures. The obtained samples were clustered using unsupervised clustering analysis to define the different KIRC subtypes with different pyroptosis expression levels. Based on this, a gene expression analysis was performed to explore the carcinogenic mechanism that is markedly related to pyroptosis. The Genomics of Drug Sensitivity in Cancer database and single sample gene set enrichment analysis (ssGSEA) algorithm were used to analyze the different treatment methods of the current prominent KIRC to determine whether pyroptosis plays a role. Finally, LASSO regression was used to screen for related genes and construct a model to predict patient prognosis. The expression levels of GSDME, CASP3, CASP4, CASP5, CHMP3, and CHMP4C were incorporated into the model construction. After verification, the prediction accuracy of the 3-, 5-, 7- and 10 years survival rates of our prognostic model were 0.66, 0.701, 0.719, and 0.728, respectively. Through the above analysis, we demonstrated the feasibility of pyroptosis in the clinical treatment of KIRC and provided novel ideas and suggestions for the clinical treatment of KIRC.

## Introduction

Kidney cancer is a common disease associated with tumors of the urinary system. According to statistics, the number of kidney cancer cases is increasing annually, having reached 74,000 by 2020 (Sung et al., 2021). Clear cell renal cell carcinoma (ccRCC/KIRC) is the main type of kidney cancer, accounting for 75% of cases (Cohen and McGovern, 2005). A particularly difficult challenge is that kidney cancer is latent, and many patients are already at the middle or advanced stage when they are diagnosed and do not respond well to surgery. Clinical data indicates that more than two-thirds of patients are already clinically metastatic at the time of discovery, and approximately 40% of patients are still at risk of recurrence after undergoing surgical treatment (Linehan and Ricketts, 2019). The development of targeted immunotherapy has filled this gap in the treatment of advanced renal cancer. However, as time passes, kidney cancer cells become resistant to targeted drugs and the uncertainty brought by immunotherapy to disease progression. Therefore, the current treatment of kidney cancer, especially KIRC, remains a challenge that must be overcome (Chen et al., 2016; Thompson et al., 2018; Xue et al., 2020). Our current focus is to find more suitable targets for the treatment, enrich the reserves of targeted drugs, and have a clear prediction of the prognosis of renal cancer.

Targeted cancer therapy has always been a popular topic in cancer research. Since the discovery of apoptosis, various forms of regulatory cell death (RCD) have been discovered and applied in the treatment of cancer (Shi et al., 2017). In the early stage, the mechanism of RCD applied to cancer is to eliminate cancer cells by upregulating the expression of genes related to apoptosis or other RCD in cancer or downregulating the signaling pathway that inhibits RCD. With further study, the pyroptosis pathway has been discovered, and its main biochemical characteristics have become increasingly clear (Fang et al., 2020). As a newly discovered type of RCD, pyroptosis is mainly caused by inflammasome bodies, and cells show characteristics of swelling, rupture of the cell membrane, and overflow of cell contents. Early studies have suggested that the caspase family regulates pyroptosis. With the discovery of the GSDM family of executive proteins involved in pyroptosis, the occurrence and specific execution process of pyroptosis became increasingly understood (Kovacs and Miao, 2017). Pyroptosis mainly relies on various membrane structures in cells to drill holes to destroy the original structure and disrupt the homeostasis of the environment, ultimately leading to cell death (Xia et al., 2020).

In this study, we focused on genes related to pyroptosis and described the role of pyroptosis in KIRC by studying the correlation between gene expression and the survival landscape of cancer patients and their clinicopathological characteristics, as well as the possibility of targeting pyroptosis to treat KIRC. At the same time, we further guided the clinical diagnosis and treatment of pyroptosis by constructing a prognostic model of KIRC.

## Result

### Widespread Genetic Mutations of Pyroptosis Related Genes

**FIGURE 1:**
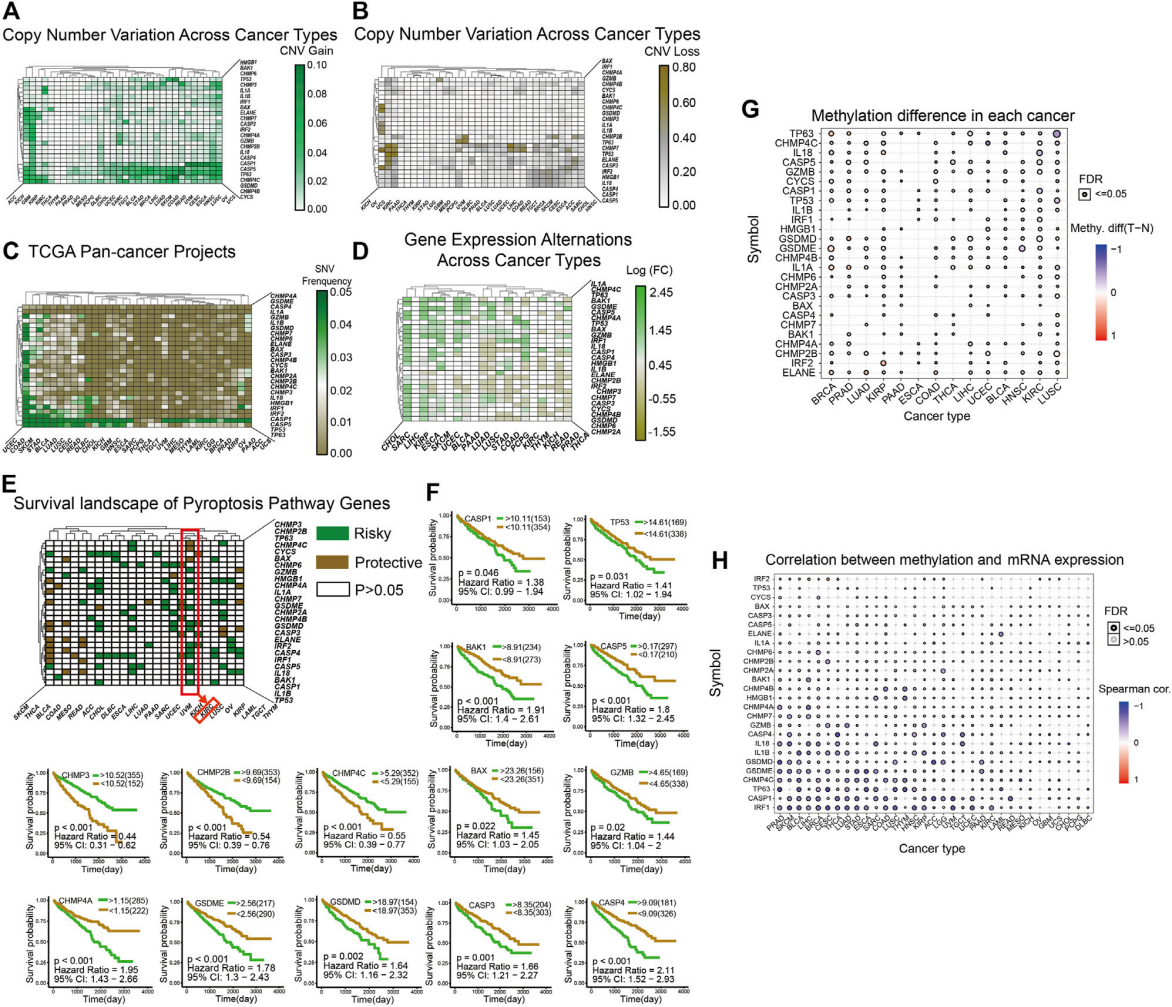
**(A–B)**: The heatmap shows the CNV mutations of pyroptosis-related genes obtained in the pan-cancer project of TCGA database. The green color in A indicates a higher degree of CNV gain, the brown color in B indicates a higher degree of CNV loss. The color bar on the right of the figure shows the specific values. **(C)**: The heatmap shows the SNV mutations of pyroptosis. The color bar on the right shows the color corresponding to the specific value: the greener the color, the closer it is to 0.05, and the browner the color, the closer it is to 0.00. **(D)**: The heatmap shows the expression levels of pyroptosis-related genes in 20 cancers, and the results are processed by log2 fold change. A transition from green to brown corresponds to a value between −1.55 and 2.45. **(E)** This heatmap shows that all pyroptosis-related genes are divided into three categories from the perspective of survival landscape: Risky genes (Green), protective genes (Brown), and no statistically significant genes (White, *p* > 0.05). The categories of genes in KIRC are marked with a red box. **(F)** This includes the survival curve analysis of all statistically significant genes in KIRC in TCGA samples. The upper right corner of each survival curve figure indicates that green represents the high expression group and brown represents the low expression group. **(G–H)** The two graphs show the different degrees of methylation in each cancer and the correlation between methylation and mRNA expression levels. The color depth of the ring on the right indicates the comparison between the *p*-value and 0.05, and the color bar indicates the degree of difference and correlation coefficient.

We used patients with cancer in The Cancer Genome Atlas (TCGA) database as sample data and heatmaps to plot the mutations of pyroptosis genes, including CNV (Figures 1A,B), SNV (Figure 1C), and mRNA expression (Figure 1D) levels. In general, genetic mutations of pyroptosis are widely present in most cancer types, except for individual cancers such as THCA, SARC, SKCM, and other non-obvious mutations in pyroptosis-related genes. Correlation analysis of gene expression and the survival landscape (Figure 1E) indicated that pyroptosis-related genes were most closely associated with KIRC, and most of the genes were risk factors in patients with KIRC. The survival curve (Figure 1F) also indicated that high expression of most pyroptosis-related genes was correlated with poor patient prognosis. Differences in methylation of pyroptosis-related genes in KIRC were extensive, and most were related to differences in mRNA expression (Figures 1G,H).

### The Three Clusters Correspond to the Expression of Pyroptosis Related Genes

**FIGURE 2:**
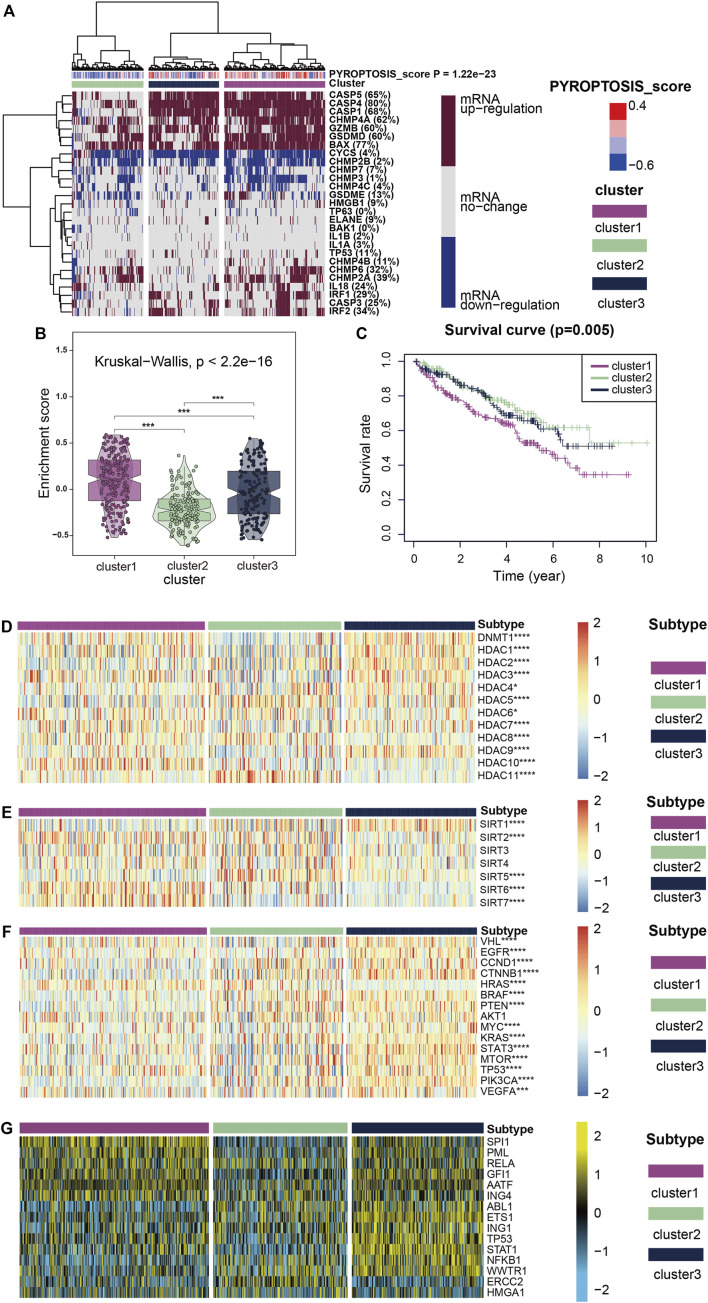
**(A)** All KIRC samples are divided into three groups according to different levels of pyroptosis-score: high expression group (cluster1), medium expression group (cluster 2) and low expression group (cluster 3. Color changes in the color bar on the right represent different values: dark red represents up-regulation of mRNA expression and dark blue represents down-regulation of mRNA expression. The closer the pyroptosis-score is to 0.4, the redder the color is, and the closer it is to -0.6, the bluer the color is. The three groups formed through cluster analysis are represented by different colors: violet represents cluster1, olive green represents cluster2, and purplish-blue represents cluster3. **(B)** The violin plots reveal the enrichment score of the three clusters, and the color differentiation was the same as in **(A) (C)** Survival curves based on the three clusters. **(D–F)** The heatmap shows the expression of the three clusters of classic oncogenes and histone deacetylation-related genes, with red represents high expression and blue represents low expression as indicated by the color block on the right. **(G)** The heatmap shows the expression levels of upstream transcriptional regulators in the three clusters, with low expression in blue and high expression in yellow, as shown in the color block on the right.

Using unsupervised cluster analysis, we classified the KIRC patient samples obtained from TCGA, and we obtained three clusters (Figure 2A). The heatmap shows that mRNA expression levels of cluster1, which represents the high expression of pyroptosis, were generally upregulated, and mRNA expression of cluster3, representing the low expression of pyroptosis, was generally upregulated. The enrichment scores (Figure 2B) and survival curves (Figure 2C) of the three clustered pyroptosis-related genes also proved this: cluster1 had a high concentration of pyroptosis-related genes and poor prognosis; cluster3 had a low concentration of pyroptosis genes and good prognosis, while cluster2 was between the two.

### Expression of Classical Oncogenes, Histone Modified Genes, and Regulon

Figures 2D,E show how two histone-related genes (HDAC and SIRT) were expressed in three clusters. The expression levels of some HDAC protein family regulatory genes such as DNMT1, HDAC1, HDAC3, HDAC7 and HDAC10 were markedly up-regulated in cluster1, while the expression level of HDAC11 was substantially down-regulated (Figure 2D). The regulation of SIRT protein family genes was interesting (Figure 2E), in addition to SIRT1 and SIRT5 showing markedly low expression in cluster1, SIRT6 and SIRT7 were highly expressed in cluster1. SIRT2, 3, and 4 showed considerably low expression in cluster3 which represents the expression level of pyroptosis genes showed little change, and at the same time in cluster1,2 was high expression, this abnormal phenomenon is thought provoking. When we focused on the expression of classical carcinogenic genes, a similar abnormal situation was observed with SIRT expression (Figure 2F). However, except for HRAS, gene expression was generally low in cluster1,2 and high in cluster3. In contrast, the expression of HRAS was high in cluster1,2 and low in cluster3.

We believe that the results of HDAC and SIRT indicate that pyroptosis mutations in cancer are closely related to histone modification. The expression of classical oncogenes lends credence to the belief that the pyroptosis mutant signaling pathway is independent of other classical cancer pathways, which has significant research value.

To further explore transcriptome differences, we obtained 15 upstream transcription factors of 27 pyroptosis-related genes from TRRUST and calculated their transcriptional regulatory network expression activity (Figure 2G) in KIRC samples. The activities of SPI1, PML, RELA, GFI1, AATF, and ING4 were considerably higher in cluster1 than in cluster2, while the activities of ERCC2 and ABL1 were markedly lower in cluster1 than in cluster2. These results suggest that epigenetically driven transcriptional networks may be important differentiators of sample clustering.

### Association Analysis of Cancer Treatment

**FIGURE 3:**
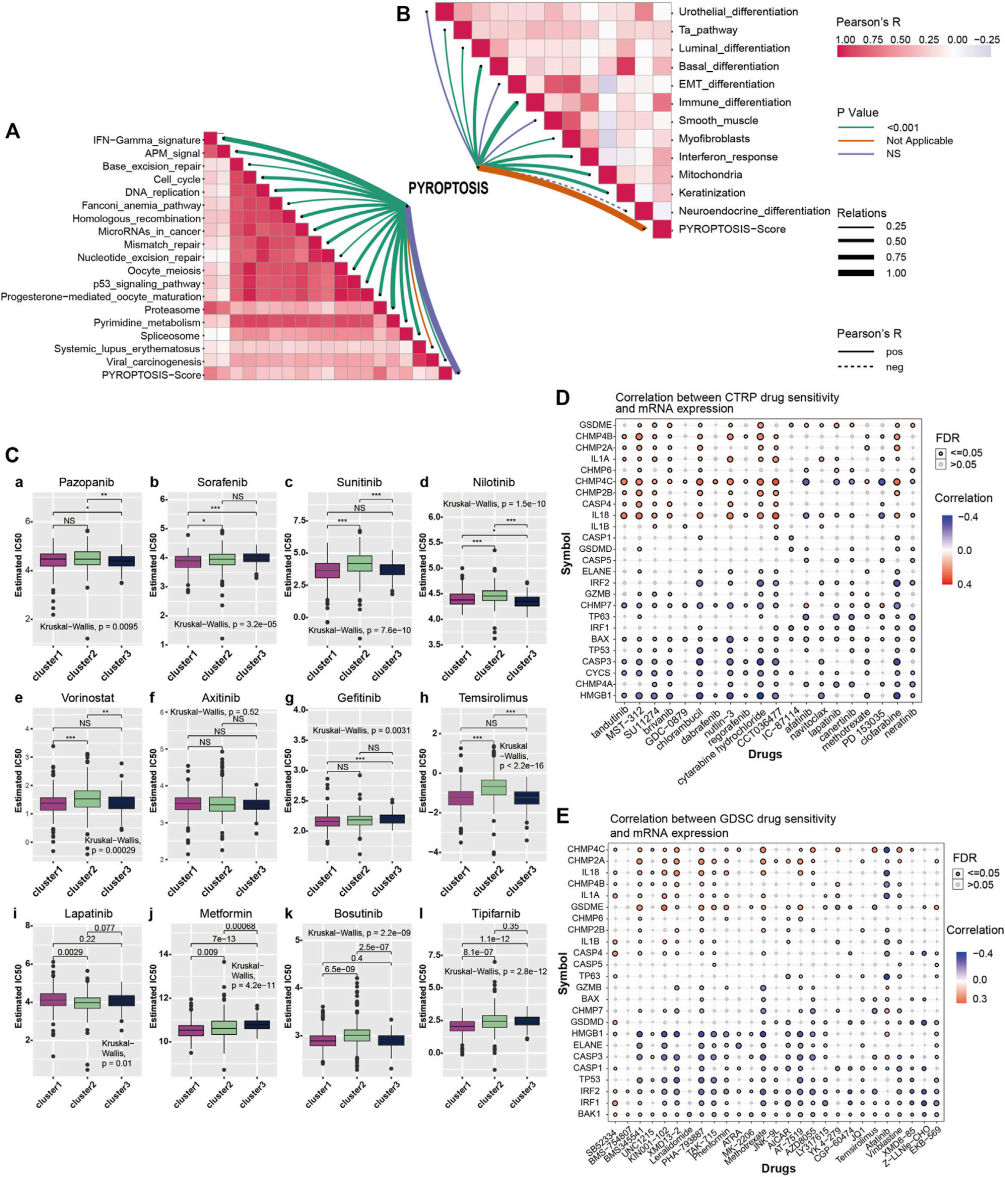
**(A)** Correlation between pyroptosis-score and immunotherapy predicted pathways. The color bar on the right indicates Pearson R that the redder the color block is, the greater the correlation coefficient is, and the more purple the correlation coefficient is. The line color shows the *p*-value, with green representing *p* < 0.001, orange and violet representing not applicable and no statistical significance, respectively. Line thickness represents correlation coefficient, ranging from 0.25 to 1.00, the solid line represents positive correlation, the dotted line represents negative correlation. **(B)** Correlation between pyroptosis-score and signature gene pathway. **(C)** The box plots show the IC50 prediction of KIRC cells treated with a common tumor-targeted drug. **(D)** The heatmap shows the correlation between the sensitivity to drugs results in various cancers obtained from the CTRP database and the mRNA expression levels of pyroptosis-related genes. **(E)** The heatmap shows the correlation between the sensitivity of drugs results in various cancers obtained from the GDSC database and the mRNA expression levels of pyroptosis-related genes.

To further explore pyroptosis treatment, we analyzed the correlation between the pyroptosis pathway and the KIRC signature pathway in KIRC and the predictive pathway of immunotherapy. The heatmap shows that many pyroptosis-related genes are closely related to many signature pathways of KIRC and immunotherapy-related pathways. In immunotherapy-related pathways (Figure 3A), the correlation between pyroptosis and other pathways was statistically significant and positively correlated except for systemic lupus erythematosus. In the KIRC signature pathway, most of the pathways except urothelial differentiation, epithelial-mesenchymal transition (EMT) differentiation, and smooth muscle were statistically significant and positively correlated with pyroptosis. Neuroendocrine differentiation was negatively correlated with pyroptosis (Figure 3B).

The Genomics of Drug Sensitivity in Cancer (GDSC) database is essential in predicting the outcomes of pharmacological trials in patients with ccRCC. Based on the expression of cell line gene profiles in the GDSC database and the support of the pRRophetic algorithm, we predicted the pharmacological effects of ccRCC cells against 12 common cancer chemotherapy agents and targeted drugs: pazopanib, sorafenib, sunitinib, nilotinib, vorinostat, axitinib, gefitinib, temsirolimus, lapatinib, metformin, bosutinib, and tipifarnib. These include many clinically common targeted drugs for ccRCC patients, such as Pazopanib, Sunitinib and sorafenib (Bukowski, 2010; Motzer et al., 2013; Qifei Wang et al., 2018), as well as metformin (Kamarudin et al., 2019), which is considered to have potential therapeutic value for many patients with cancer. The results of drug IC50 prediction analysis showed that the predicted IC50 value of most targeted drugs for cluster1 was significantly lower than that of cluster2, indicating that patients with KIRC and high expression of pyroptosis were more sensitive to these conventional targeted drugs (Figure 3C). Thus, the application of these targeted drugs to treat patients with KIRC and high pyroptosis expression is of particular significance. From the results, we observed that common clinically targeted drugs for renal cancer at present: sunitinib, sorafenib, and temsirolimus have high sensitivity to ccRCC cells with low pyroptosis expression, which also indicates that the pyroptosis pathway may have a guiding significance for the development of targeted drugs for ccRCC.

### Analysis of Immune Infiltration and Immune Checkpoint Block

**FIGURE 4:**
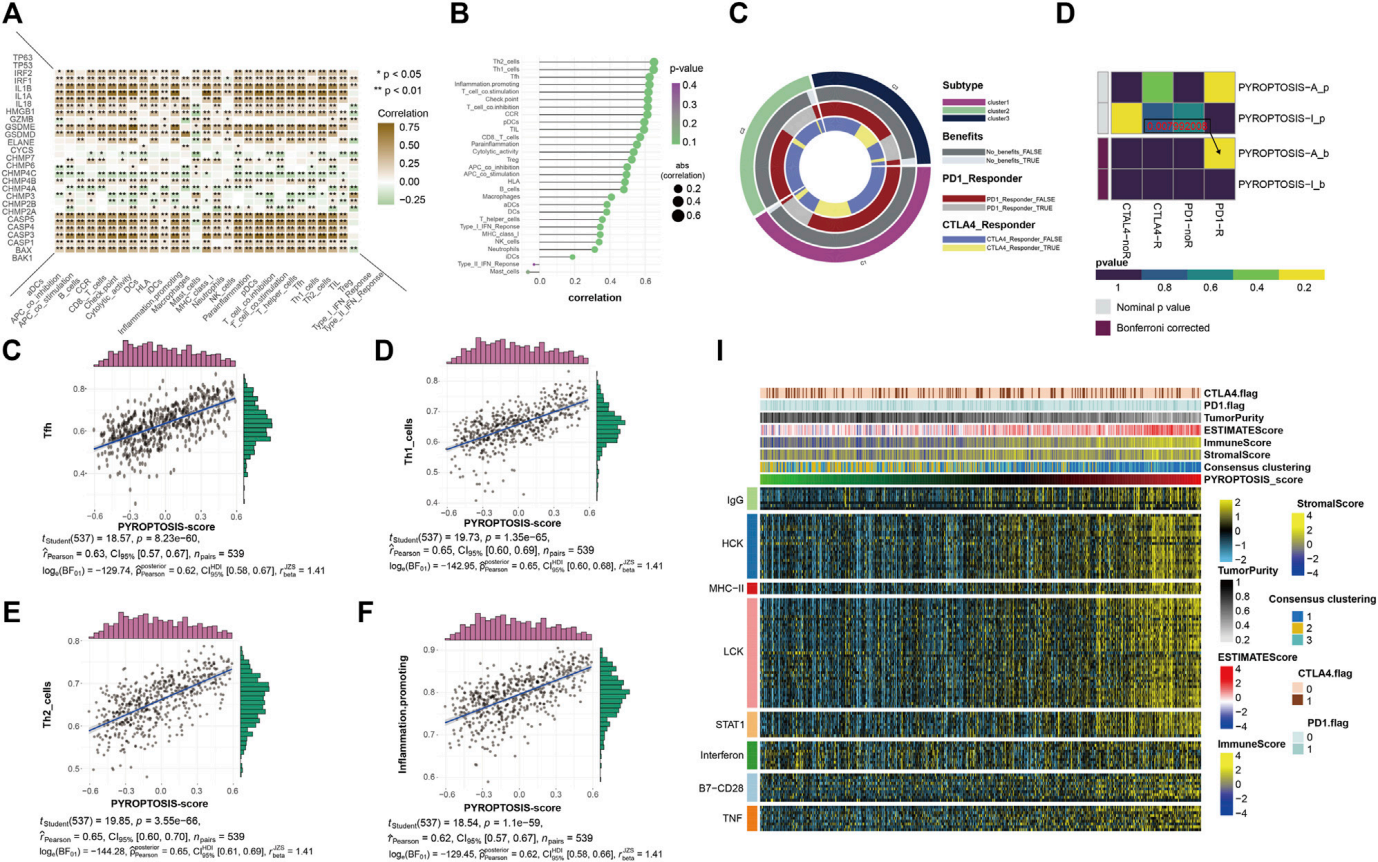
**(A)** The heatmap shows the correlation between all pyroptosis-related genes and various immune infiltration-related indicators. The color bar on the right shows that the closer to brown, the greater the positive correlation, and the closer to green, the greater the negative correlation. * representative *p* < 0.05, ** represents *p* < 0.01. **(B)** The bubble plot shows the degree of correlation. The bubble size represents the correlation size from 0.2 to 0.6, and the color represents the *p*-value from 0.1 to 0.4. **(C–F)** The four scatter plots respectively show the correlation between pyroptosis- score and Tfh, Th1 cell, Th2 cell, and inflammation-promotion. **(G)** The ring plot shows the predicted efficacy and benefit of three clusters of different immune checkpoint blocking therapies. **(H)** The heatmap shows the results of predicting the *p*-values obtained by comparing pyroptosis-active and pyroptosis-inactive samples using PD-1 and CTLA4 treatment, and the comparison results of the *p*-values after Bonferroni correction. **(I)** To identify the meta-genes of major expression vectors associated with immunotherapy, we screened the samples for 113 genes associated with 8 inflammatory factors. The heatmap shows the expression of these 113 genes and other immunotherapy-related scores in the KIRC sample.

The application of PD-1 therapy in the treatment of these patients has excellent prospects. The results of this section confirm that pyroptosis has value in tumor immunotherapy, which is consistent with the results of previous studies. The heatmap shows a quantified correlation between immune cell infiltration and pyroptosis (Figure 4A). The pyroptosis pathway has a strong correlation with the immune infiltration of patients with KIRC. The bubble plot shows the correlation sequence between immune infiltration-related cells and pyroptosis (Figure 4B). We selected the first four immune cells with strong correlation to demonstrate their correlation, and they were positively correlated with pyroptosis (Figures 4C–F). The heatmap showed that the response of the pyroptosis-active group to immune checkpoint block therapy after the correction was statistically significant (*p* = 0.007992008) (Figure 4H). This indicates that patients with KIRC and high expression of pyroptosis-related genes are likely to respond to immune checkpoint blockade, and PD-1 or treatment with CTLA-4 has application prospects for these patients. Extension of the sample data to the three previously obtained clusters showed a similar pattern (Figure 4G). Finally, to understand the effects of pyroptosis on various types of immune systems, we used heatmaps to show the correlation between different samples in the identified different metagenes and sorted the samples according to the pyroptosis-score size obtained by cluster analysis (Figure 4I). The heatmap shows that as the pyroptosis score increases, the expression level of each metagene in the sample also gradually increases. It can be considered that the pyroptosis pathway has a more extensive impact on the immune system.

### Prediction Model Based on Patient Clinical Information and Gene Expression

**FIGURE 5:**
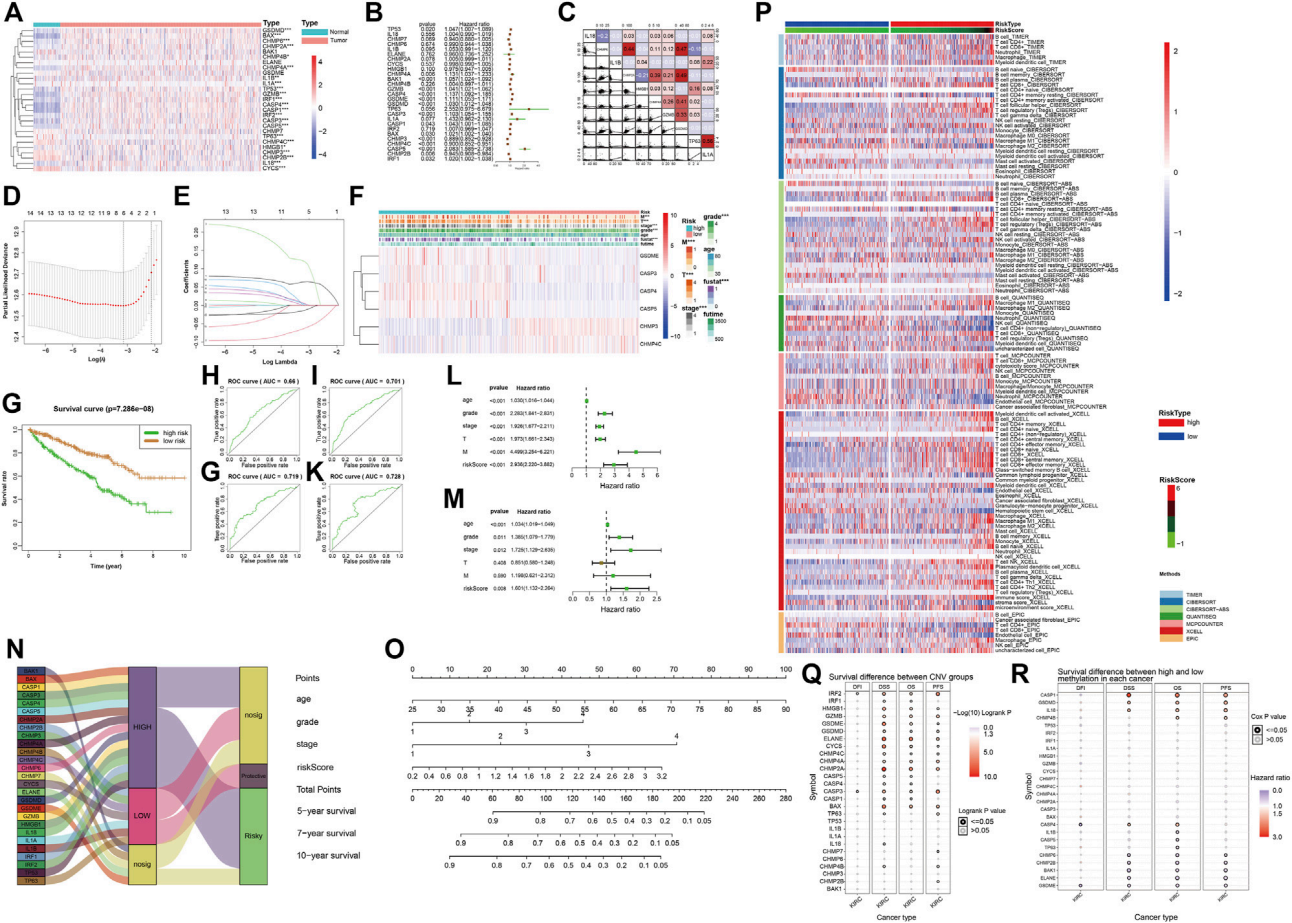
**(A)** The heatmap shows the expression of pyroptosis-related genes in the tumor group (pink) and the normal group (light blue). The color bar on the right indicates that the closer to red, the more up-regulated the expression, the closer to blue, the more down-regulated the expression. (*: *p* < 0.05, **: *p* < 0.01, ***: *p* < 0.001) **(B)** The forest map shows the hazard ratio of different pyroptosis-related genes in KIRC. **(C)** The co-expression relationship between 10 pyroptosis-related genes with significant statistical significance, their regression relationship is displayed with scatter plots, the correlation coefficient is represented by color, a positive correlation is red, a negative correlation is blue, the deeper the color represents the greater correlation. **(D–E)** Six variables (genes) were screened out by LASSO regression to construct the prediction model. **(F)** The heatmap shows differences in the expression of the six selected genes and clinicopathological features between the high- and low-risk groups with a risk-score cut-off =0.5. (*: *p* < 0.05, **: *p* < 0.01, ***: *p* < 0.001) **(G)** Survival curves of high-risk and low-risk groups. **(H–K)** The ROC curve of survival prediction at 4-time points shows the authenticity of the prognostic model, from left to right: top row: 3 years, 5 years, bottom row: 7 years, 10 years. In general, AUC> 0.7 is considered to be true. **(L)** Univariate Cox regression analysis. **(M)** Multivariate COX regression analysis. **(N)** The Sankey plot shows the classification of these pyroptosis-related genes after our analysis: the level of expression in KIRC tumor tissues and the role of cancer suppressor or carcinogenic in KIRC. **(O)** The nomogram of the prognostic model. **(P)** Heatmap for immune responses based on different algorithms among the high- and low-risk groups. Different algorithms are represented by different colored area bars. **(Q)** Correlation between CNV mutations of different pyroptosis-related genes and survival coefficient in KIRC. **(R)** Correlation between methylation of different pyroptosis-related genes and survival coefficient in KIRC.

The heatmap and forest map show the differences in the expression of 27 genes in normal and tumor tissues and their hazard ratios for diseases (Figures 5A,B). We randomly selected 10 genes with statistically significant effects on KIRC (*p* < 0.05) for co-expression analysis (Figure 5C), and the results showed that they had a strong co-expression relationship. The number of independent variables in the KIRC sample is large. When using 27 gene expressions to construct the prediction model, model distortion inevitably occurs owing to excessive variables. To reduce the interaction between variables, LASSO regression was used using its regularization mechanism to punish variables, eliminate genes with strong collinearity, reduce the number of variables, and prevent overfitting (Figures 5D,E). Based on the selected genes, we calculated the risk scores of the samples. Eventually, we obtained six genes for building the model: GSDME, CASP3, CASP4, CASP5, CHMP3, and CHMP4C. According to the risk score values obtained, the samples were divided into two groups with high and low risk, and their clinicopathological features (Figure 5F) and survival landscape (Figure 5G) were compared. GSDME, CASP3, CASP4 and CASP5 are highly expressed in the high-risk group, while CHMP3 and CHMP4C have a lower expression in the high-risk group. Receiver operating characteristic (ROC) graphs were then drawn to calculate the AUC values. The results showed that, except for 3- (Figure 5H), 5- (Figure 5I), 7- (Figure 5J) and 10-years (Figure Fig5K) were all authentic. Univariate COX regression (Figure 5L) and multivariate COX regression (Figure 5M) confirm that the risk score could be used as a prognostic factor (*p* < 0.05). The Sankey plot (Figure 5N) was used to show the final flow of the results obtained after the analysis of the initial 27 genes, and the column diagram (Figure 5O) was used to visualize the prediction model. In the subsequent LASSO regression model building process, we demonstrated the correlation between the risk score and immune cells in various algorithms according to the order of the risk score after obtaining the sample risk score (Figure 5P).

### Supplementary Results for GSCA

We obtained the correlation between pyroptosis-related genes and KIRC from the GSCA website. The results obtained from GSCA related to pyroptosis pan-cancer analysis (Figures 1G,H), drug prediction (Figures 3D,E), and immunotherapy (Figures 5Q, R) were consistent with the results we analyzed.

## Discussion

With improvements in the tumor gene spectrum, cancer therapy has evolved from early radiotherapy and chemotherapy to targeted therapy, immunotherapy, and other precision therapies (Blagosklonny, 2005). These methods mainly focus on preventing cancer cell biosynthesis and reducing their proliferation and metastasis. With the research progress, scientists have found that the expression of genes regulating cell death in cancer cells is mostly inhibited (Strasser and Vaux, 2020). This leads to RCD in cancer cells not being carried out. Therefore, the induction of cancer cell death by regulating RCD-related genes has become a focus of research (Garg and Agostinis, 2017). Pyroptosis is a type of RCD usually caused by inflammatory cells. Its main feature is cell expansion until the cell membrane ruptures, and the content overflows, causing a strong inflammatory response. The occurrence of pyroptosis depends on the caspase and GSDM protein families. After the GSDMs protein is cleaved by activated caspase, the GSDM n-terminal (GSDM-nt) is released, bound to the cell membrane, and penetrates the cell membrane, resulting in changes in cell osmotic pressure, cell swelling, and rupture (Tang et al., 2019).

In our study, we first evaluated the role of pyroptosis-related genes in cancer using a pan-cancer analysis. In pan-cancer analysis, pyroptosis has been correlated with most cancers. In cancers such as UCEC, COAD, and SKCM, more than half of the genes in the entire gene set have mutations. In addition to SARC, PAAD, SKCM, THYM, and other cancers, there was no significant change in expression, and the expression levels in 20 types of cancer samples were changed. This indicates that pyroptosis is an important factor in the occurrence and progression of cancer. In the survival landscape analysis of samples, we noticed that most of the pyroptosis-related genes in the KIRC samples had a risk-associated effect. These genes are roughly classified and can be divided into caspase regulatory genes (Kesavardhana et al., 2020) (CASP1, 3, 4, 5), GSDM regulatory genes (Kovacs and Miao, 2017) (GSDMD, GSDME), apoptosis-related protein regulatory gene (Bouillet and Strasser, 2002) (BAX, BAK), and CHMP family genes (Tsang et al., 2006). Subsequently, through unsupervised cluster analysis, we divided the KIRC samples into three clusters (cluster1, cluster2 and cluster3) and conducted a follow-up analysis.

Our results showed that caspase and GSMD family proteins, as well as apoptosis-related and CHMP family proteins involved in pyroptosis, were correlated with the occurrence and progression of KIRC. In the survival landscape analysis, we noted that most pyroptosis-related genes in the KIRC samples play a risk-associated role. Based on the mechanism of pyroptosis, many researchers regard the activation of pyroptosis in cancer as a new method to study cancer treatment. However, our study concluded that the overall high expression of pyroptosis-related genes in KIRC is not beneficial for the survival of patients with cancer. To explore this abnormal phenomenon and clarify the specific mechanism by which pyroptosis affects the survival rate of patients with KIRC, we analyzed the expression of histone modification and classical carcinogenic genes in the expression matrix of the three clusters. These results showed a correlation between pyroptosis and histone modifications. We speculated that this might be one of the reasons why pyroptosis plays a risk-associated role in KIRC; pyroptosis reduces gene stability by affecting histone modification, leading to the progression of KIRC. There have been no detailed studies on the connection between pyroptosis and histones in recent years, and only some studies have speculated on the relationship between the two mechanisms based on the correlation between family proteins (Chen et al., 2018; Magupalli et al., 2020). Therefore, we believe that it is necessary to conduct in-depth studies on the specific relationship between pyroptosis and histones, which is of great help in the treatment of various cancers, including KIRC, and other diseases related to pyroptosis. In this regard, we believe that this indicates that pyroptosis is independent of other carcinogenic mechanisms in KIRC and is unique, which also highlights the importance of studying the effects of pyroptosis on KIRC.

At present, KIRC treatment mainly involves targeted drugs and immune checkpoint blocking therapy (Caliskan et al., 2020). Subsequently, we conducted a variety of analyses for the treatment of KIRC, including drug and immune predictive analyses. As can be seen from the drug prediction results, in most targeted drugs, different clusters have different drug sensitivities, which indicates that the drug mechanism of KIRC and pyroptosis mechanism currently common targeted drugs overlap, and the pyroptosis mechanism contributes to targeted drug therapy, so it revolves around pyroptosis mechanisms that have certain clinical value in developing new targeted drugs. After determining the correlation between immune cells and pyroptosis, we found that pyroptosis was markedly beneficial in the response of patients to PD-1 treatment by judging the response of different pyroptosis expressions to PD-1 and CTLA-4 immune checkpoint blockade. This means that patients with high pyroptosis expression responded better to PD-1 treatment. The application of PD-1 therapy in treating these patients has excellent application prospects (Miao et al., 2018). The results confirmed that pyroptosis has value in tumor immunotherapy, which is consistent with the results of the previous studies (Wang et al., 2020). Pyroptosis is also associated with other immune system components in patients with KIRC, proving that pyroptosis is of high value in immune infiltration and inflammation-related immunotherapy (Aachoui et al., 2013).

Using the clinicopathological information from TCGA, we constructed a prediction model for pyroptosis, and classified all 27 pyroptosis-related genes, and found that, except for a small number of genes with high expression, such as IL1A, IL-1B, and TP63, which play a protective role on KIRC, most of the other genes, including Casp3-45, GSDME, and CHMP3 4C screened by LASSO regression, were classified as risk genes. ROC curves confirmed that our predictions for 5-, 7-, and 10-years were true and accurate, which indicates that our model has clinical prediction ability. When exploring the molecular mechanism of pyroptosis, we found an interesting finding. The GSDME protein of pyroptosis can not only drill holes in the cell membrane but also in the nuclear membrane (Majno and Joris, 1995; D’Arcy, 2019). In an accurate sense, GSDME protein is a biofilm drilling protein. We speculate that this situation can explain the abnormal phenomenon in KIRC: patients with high expression of pyroptosis can produce a large number of GSDME proteins in cancer cells and drill holes in many biofilms in cells. Drilling of the cell membrane can be repaired by cancer cells themselves, but nuclear membrane drilling disturbs internal gene stability and increases mutation probability. This will accelerate cancer progression and lead to a low survival rate in patients. However, the application of some drugs increases the efficacy of GSDME in different ways, disrupts the balance between biofilm damage and repair, and directly destroys cancer cells, and thus still has an excellent therapeutic effect on patients (Hu et al., 2020; Zhang et al., 2020; De Schutter et al., 2021; Ibrahim et al., 2021).

## Material and Method

### Data Acquisition and Preliminary Analysis: The Cancer Genome Atlas Database

In the early stages, we used GSEA software to obtain 27 genes closely related to pyroptosis from the REACTOME database (Jassal et al., 2020) (https://reactome.org/). The set of cellular biomolecular pathways in the REACTOME database helped to quickly and accurately identify the pathways and genes associated with pyroptosis. After identifying pyroptosis-related genes, sample data of all cancers, including KIRC and control groups, were obtained using the R/Bioconductor package TCGAbiolinks (Colaprico et al., 2016) from the pan-cancer project in TCGA database (Tomczak et al., 2015) (https://portal.gdc.cancer.gov/), including basic information of samples, gene expression, clinicopathological characteristics. The TCGA database currently contains various clinical and gene expression data of more than 20,000 patients with more than 30 cancer types, which is of great help for the analysis of specific genes or pathways in pan-cancer. RNA-seq cohort of KIRC included 72 normal and 539 cancer samples. The Perl language was used to process the data and analyze the correlations between them. The gene expression data of 72 para-carcinoma tissue samples and 539 cancer samples obtained from TCGA, as well as the clinical data of the latter, were included in the study without artificial screening or elimination. In the analysis of clinical and pathological information, we decided to screen out samples with missing information on tumor, node, metastasis (TNM), stage, grade, and survival time of fewer than 30 days from the 539 ccRCC patient samples obtained from TCGA. At the same time, we used K-nearest neighbor (KNN) to fill in the missing gene expression values of part samples.

### Grouping of Data: Cluster Analysis

We used the GSVA algorithm in R (Hänzelmann et al., 2013) to calculate the enrichment score of pyroptosis-related genes and then screened the genes with differences between samples and determined whether they were highly expressed, poorly expressed, or not differentially expressed (halfwidth = 0.025). Based on the difference in signature gene expression, cluster1 (high expression), cluster2 (low expression) and cluster3 (medium expression) were obtained by clustering analysis of these samples using the Ward.D algorithm. We then constructed violin plots of signature gene enrichment scores and survival curves in the three clusters to verify the differences among the three clusters.

Differential analysis of the three clusters: classical oncogenes and histone modifications

Based on the three clusters obtained, we used R to draw a heatmap (pheatmap package) to show the differences in gene expression of various types. Among them are the classic oncogenes in KIRC: VHL, EGFR, TP53 and 15 other common oncogenes (Jonasch et al., 2021). They have an impact on the proliferation of cancer cells, metastasis, and generation of blood vessels that supply cancer tissue. Chemical modification of histones that make up chromosomes is considered an important factor in the cancer development. Differences in the expression of two genes that have important effects on histone modification, the SIRT(Jeh et al., 2017) gene family and the HDAC (Haberland et al., 2009) gene family, in the three clusters are also shown in the heatmap. The upstream transcription factor of pyroptosis was obtained from TRRUST (https://www.grnpedia.org/trrust/)(Han et al., 2018), the transcriptional regulatory network was constructed using the RTN package in R (Groeneveld et al., 2019), and regulon activity was calculated.

### Cancer Treatment: Multi-Pathway Correlation Analysis and Drug Therapy Prediction

To explore the multiple possibilities of KIRC treatment, the GSVA algorithm was used to calculate the enrichment degree of pyroptosis-related genes in the KIRC signature and immunotherapy prediction pathways, and the results were expressed using two autocorrelation heatmaps. The GDSC database provided a large number of drug sensitivity test results for cancer cells for different targeted drugs. Using the GDSC database (https://www.cancerrxgene.org/) (Yang et al., 2012), we could quickly identify targeted drugs with apparent therapeutic effects on KIRC. Using the pRRophetic predictable function provided by the pRRophetic package in R (Geeleher et al., 2014), a ridge regression model was constructed to predict the IC50 of drugs according to the cell line and TCGA gene expression profiles in GDSC. Several classic and novel targeted drugs have been selected to treat KIRC tumor cells, including pazopanib, sorafenib, sunitinib, nilotinib, vorinostat, axitinib, gefitinib, temsirolimus, lapatinib, metformin, bosutinib, and tipifarnib. Using the GDSC database, we compared the therapeutic ability of several common tumor-targeting drugs against cancer cells in three clusters, and the results are represented in IC_50_.

### Immune Checkpoint Inhibition (Haanen and Robert, 2015): Programmed Cell Death Protein 1 and CTLA-4

Immune infiltration-related indicators of KIRC represent the sensitivity of KIRC to immunotherapy (Zhang et al., 2019). We use the ssGSEA algorithm in R (Subramanian et al., 2005; Xiao et al., 2020) to quantify the level of immune cell infiltration, using Spearman rank correlation to calculate the correlation between genes and levels of immune cell infiltration. Finally, we obtained the correlation between pyroptosis-related genes and immune infiltration-related indicators and drew a heatmap to display the results. At the same time, scatter plots were used to show the regression relationship between several important immune infiltration indicators and pyroptosis. We drew a bubble plot to show the correlation coefficients of the pyroptosis score and various immune infiltration indicators. To help us better analyze, we selected cluster1 (high expression) and cluster2 (low expression) for comparison. Using the TIDE algorithm (http://tide.dfci.harvard.edu/login/), we calculated the response of pyroptosis-active and pyroptosis-inactive KIRC patients to the two immune checkpoint blocking therapies, PD-1 (Chamoto et al., 2017) and CTLA-4 (Mitsuiki et al., 2019). We performed Bonferroni correction on the *p*-values obtained and showed the results with a heatmap.

### Reduction Feature: Metagenes

Based on the assumption that the expression of a large number of genes is highly interdependent, we applied unsupervised clustering for feature reduction. Cluster analysis is based on the association between the expression of a set of genes in different cell types in a sample and the differentiation programs/pathways associated with a particular expression profile. We excluded genes that did not show correlations with other genes above a certain threshold (0.7) and finally screened clusters containing at least 10 elements, totaling 112 probesets, to identify metagenes (major vectors).

Filtering of variables and building a prediction model: Univariate and multivariate COX regression and LASSO regression

Using the glmnet and survival package in R, we used LASSO regression to construct the model (Engebretsen, 2019). The risk score was calculated using the best cut-off value, and the samples were divided into high-risk and low-risk groups. After the number of variables (genes) was reduced to six by LASSO regression, the risk score (Risk score = ∑n i = 1 (Expi*Coei), N, Coei, and Expi represent gene number, the regression correlation coefficient obtained by LASSO regression analysis, and gene expression level, respectively.) and other clinical information of the samples were used to construct a prediction model. Test samples were used to verify the model, and the authenticity of the prediction model at different times (3-, 5-, 7-, and 10-years) was determined by drawing ROC plots. Finally, the results are displayed in the form of a nomogram. We then used a heatmap to show the correlation between the risk-score values obtained from LASSO regression and different immune cells in various algorithms, including TIMER, CIBERSORT, Cibersort-ABS, QUANTISEQ, MCPCOUNTER, XCELL, and EPIC.

### Verification of Results: Gene Set Context AnalysisLite

GSCALite (Liu et al., 2018) is a database used for the analysis of gene set cancers related to mRNA expression, mutation, immune infiltration, and drug resistance. GSCA integrated more than 10,000 multidimensional genome data of 33 cancers from TCGA and more than 750 small-molecule drugs from GDSC and CTRP. The ImmuCellAI algorithm was used to analyze the genomes of 24 immune cells. We used this website for gene set genome (expression level, CNV, SNV, and DNA methylation) and immune genome analysis. We obtained the results of the correlation analysis between genomic expression and mutation level and samples of survival and immunity of patients with KIRC from the GSCA website.

## Data Availability

The datasets presented in this study can be found in online repositories. The names of the repository/repositories and accession number(s) can be found in the article/supplementary material.

## Glossary

RCD: regulatory cell death
KIRC: kidney renal clear cell carcinoma
RCC: renal cell carcinoma
ACC: Adrenocortical carcinoma
BLCA: Bladder Urothelial Carcinoma
BRCA: Breast invasive carcinoma
CESC: Cervical squamous cell carcinoma and endocervical adenocarcinoma
CHOL: Cholangiocarcinoma
DLBC: Lymphoid Neoplasm Diffuse Large B-cell Lymphoma
ESCA: Esophageal carcinoma
GBM: Glioblastoma multiforme
HNSC: Head and Neck squamous cell carcinoma
KICH: Kidney Chromophobe
KIRP: Kidney renal papillary cell carcinoma
LAML: Acute Myeloid Leukemia
LGG: Brain Lower Grade Glioma
LIHC: Liver hepatocellular carcinoma
LUAD: Lung adenocarcinoma;
LUSC: Lung squamous cell carcinoma
MESO: Mesothelioma
OV: Ovarian serous cystadenocarcinoma
PAAD: Pancreatic adenocarcinoma
PCPG: Pheochromocytoma and Paraganglioma
PRAD: Prostate adenocarcinoma
READ: Rectum adenocarcinoma
STAD: Stomach adenocarcinoma
STES: Stomach and Esophageal carcinoma
UCS: Uterine Carcinosarcoma
UVM: Uveal Melanoma
THCA: thyroid carcinoma
THYM: thymoma
UCEC: uterine ccorpus endometrial carcinoma
SKCM: skin cutaneous melanoma
COAD: colon adenocarcinoma
TCGA: the cancer genome atlas
GSCA: Gene Set Context Analysis
CNV: the copy number variation
SNV: the single nucleotide variation
LASSO: least absolute shrinkage and selection operator
GSEA: gene set enrichment analysis;
CTLA-4: cytotoxic t-lymphocyte-associated protein 4
PD-1: programmed cell death protein 1
SPI1: Hematopoietic Transcription Factor PU.1
PML: Promyelocytic leukemia protein
RELA: RELA Proto-Oncogene, NF-KB Subunit
GFI1: Growth Factor Independent 1 Transcriptional Repressor
AATF: Apoptosis Antagonizing Transcription Factor
ING4: nhibitor Of Growth Family Member 4
HDAC: istone Deacetylase
SIRT: Sirtuin
SARC: Sarcoma
HMGB1: High Mobility Group Box 1
BAK: BCL2 Antagonist/Killer
BAX: BCL2 Associated X, Apoptosis Regulator
CHMP: Charged Multivesicular Body Protein
IL: Interleukin
IRF1: Interferon Regulatory Factor 1
ELANE: Elastase, Neutrophil ExpressedCASP: Caspase
GSDM: Gasdermin GZMB: Granzyme B
TP63: Tumor Protein P63
TP53: Tumor Protein P53
CYCS: Cytochrome C
