# Supplementary figures and images for: Potential Application of Pyroptosis in Kidney Renal Clear Cell Carcinoma Immunotherapy and Targeted Therapy
Source: Front Pharmacol. 2022 Jun 15;13:918647. doi: 10.3389/fphar.2022.918647 (PMC9252305; doi:10.3389/fphar.2022.918647)

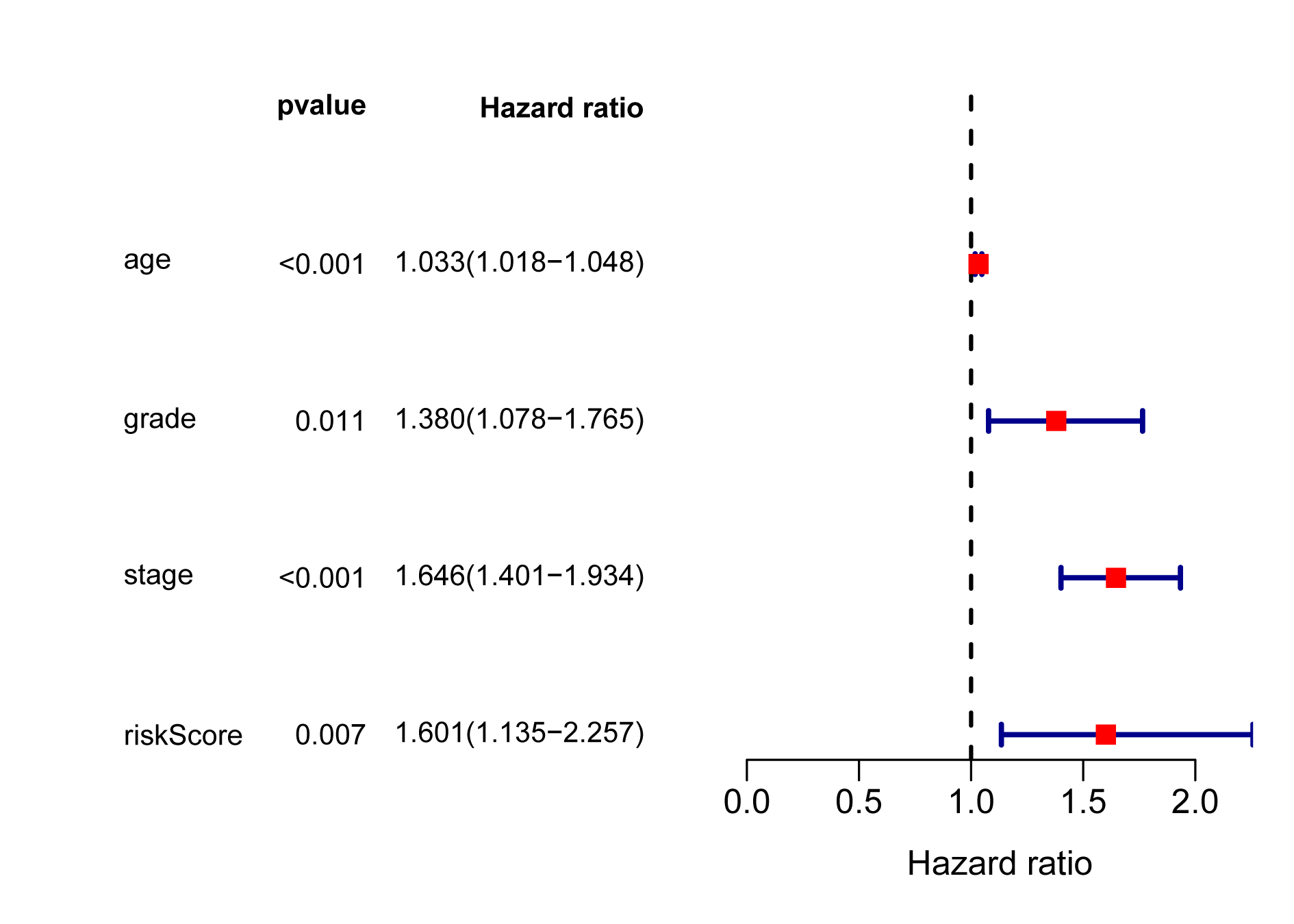

Supplement: Supplementary file 3 [file Image6.TIF]

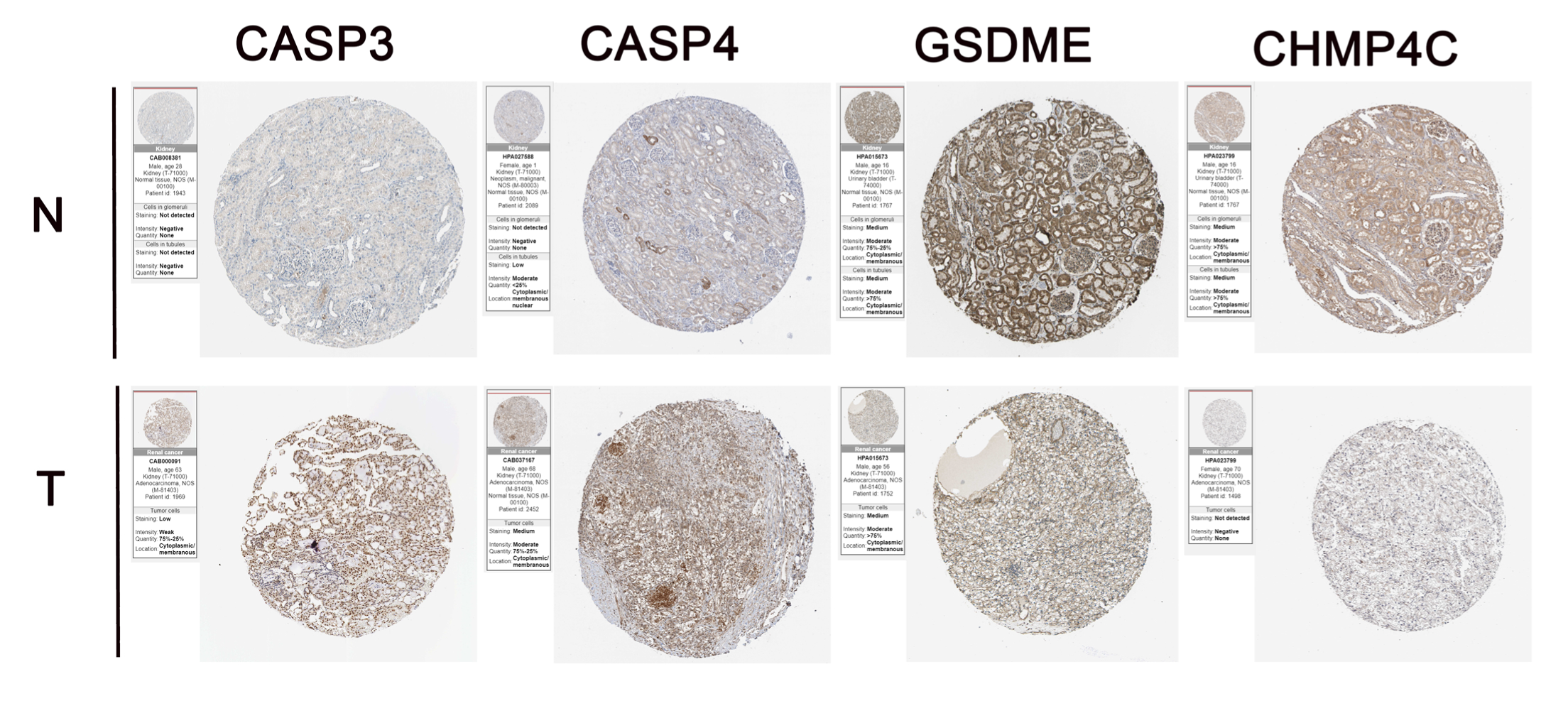

Supplement: Supplementary file 5 [file Image3.TIF]

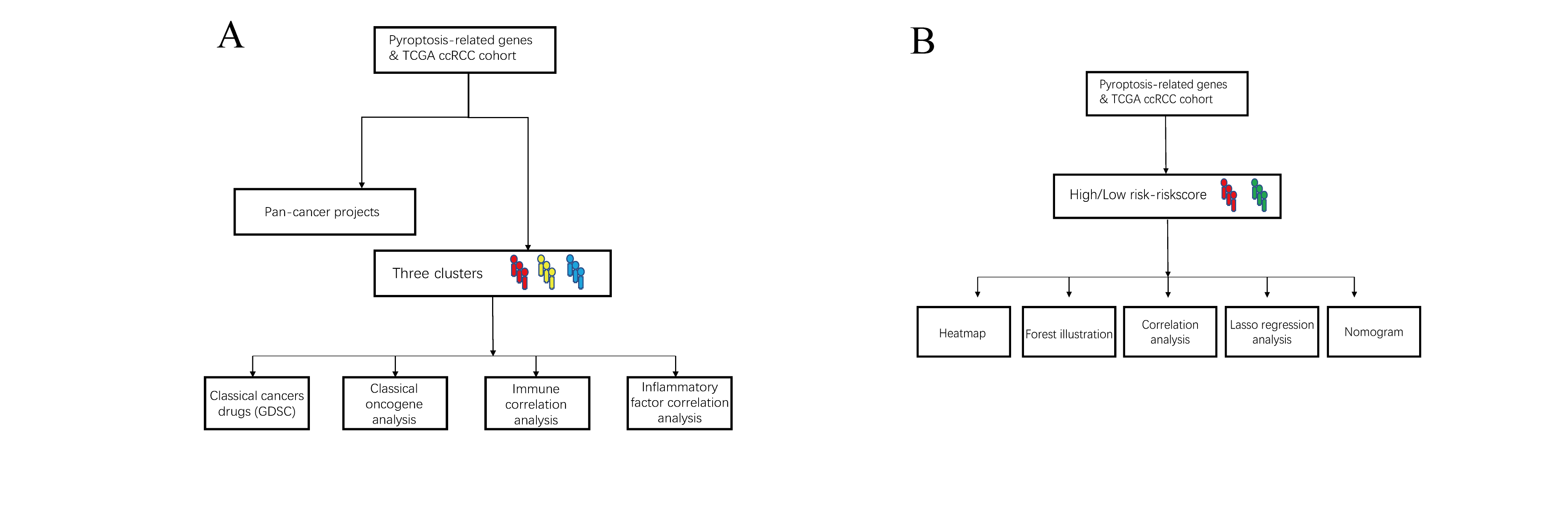

Supplement: Supplementary file 6 [file Image4.TIF]

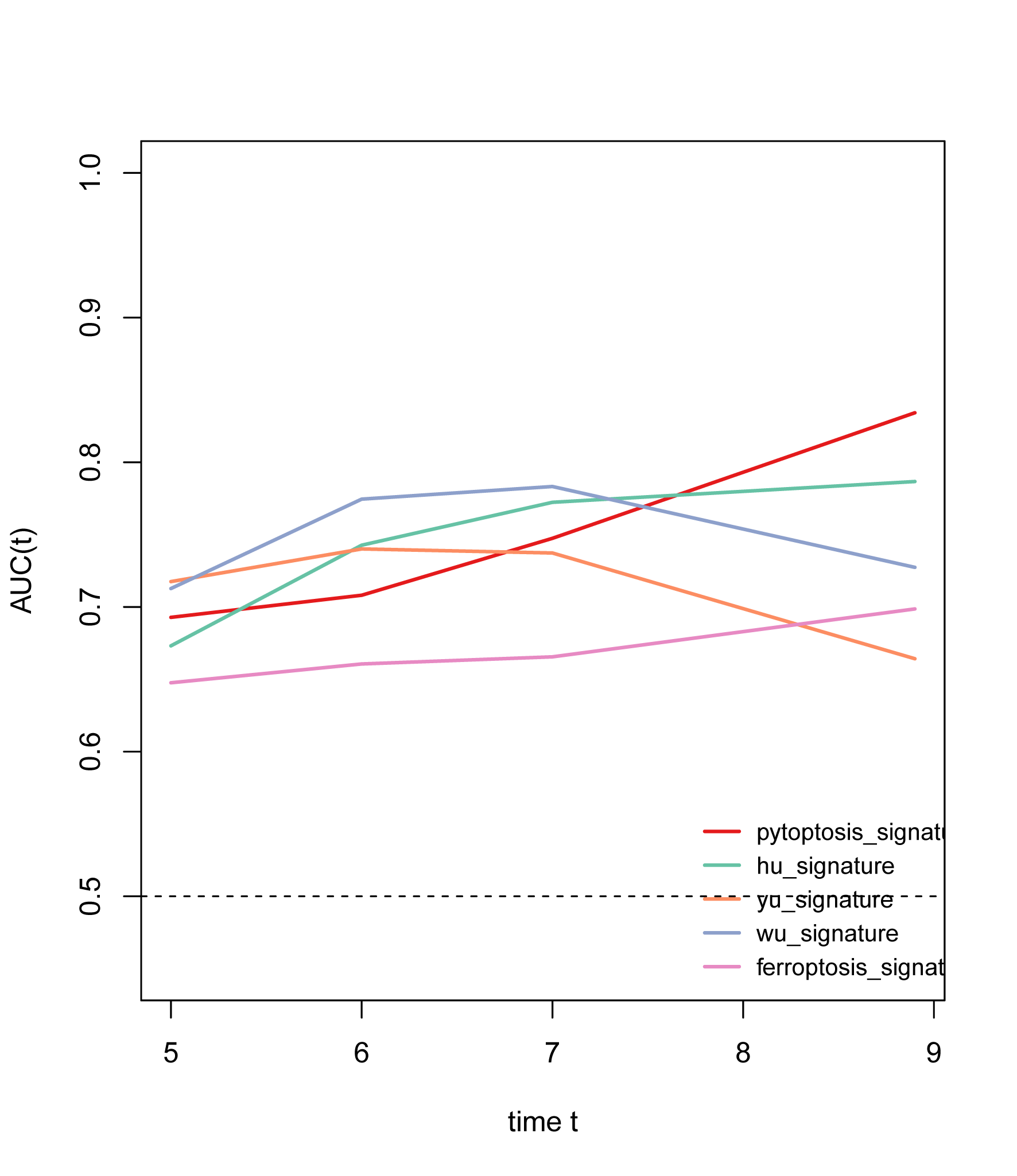

Supplement: Supplementary file 7 [file Image2.TIF]

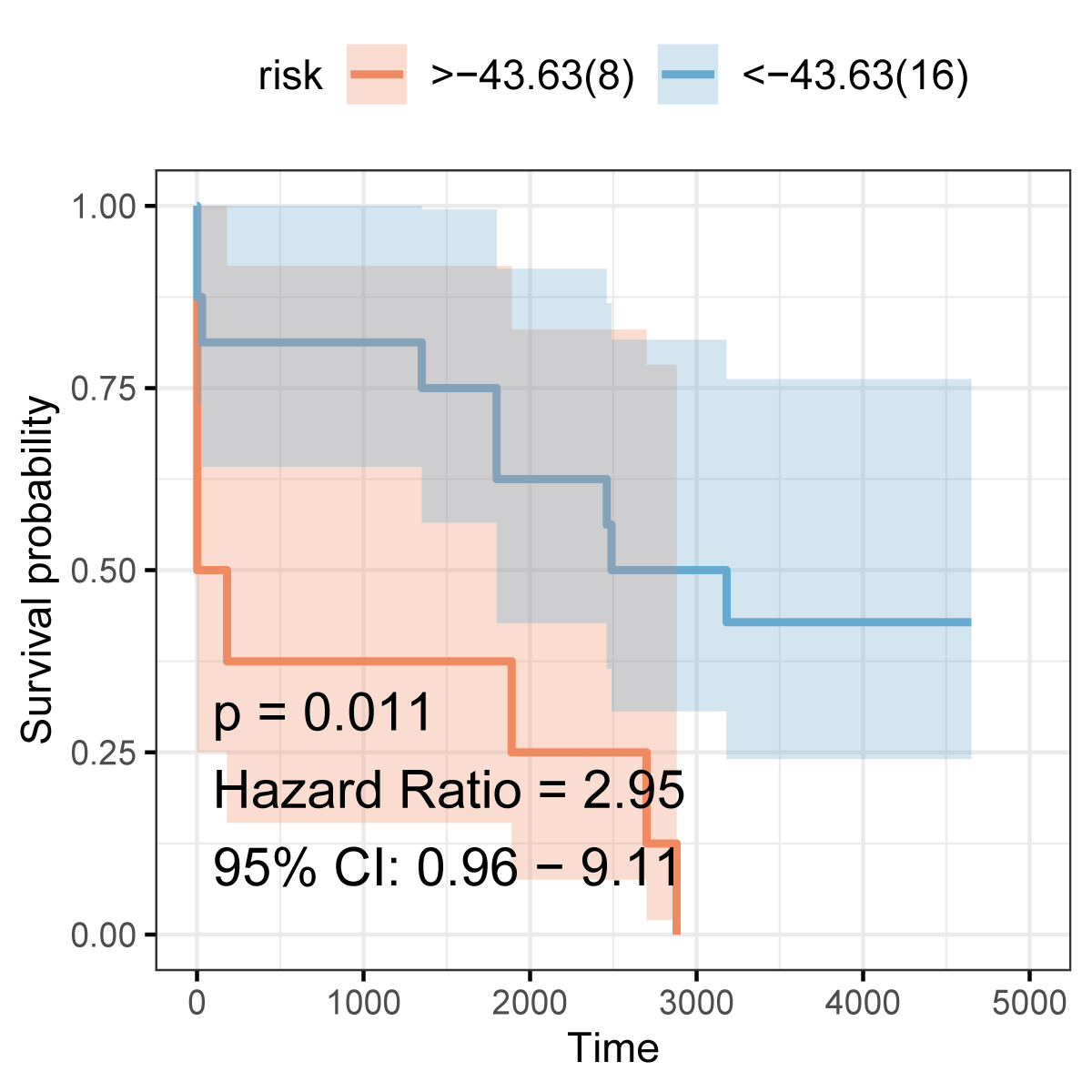

Supplement: Supplementary file 8 [file Image1.TIF]

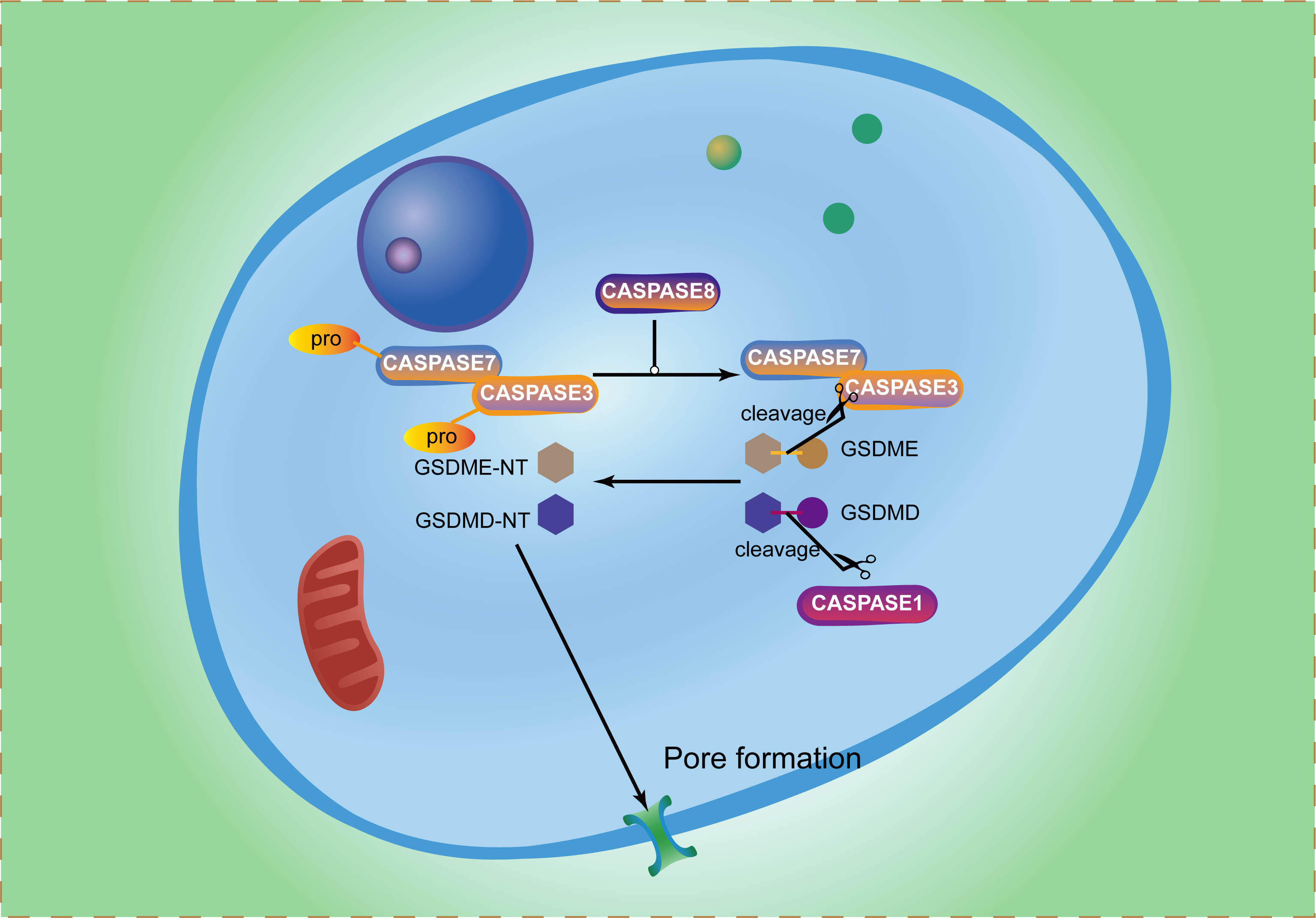

Supplement: Supplementary file 13 [file Image5.TIF]
